# High‐precision localization of radiation isocenter using Winston‐Lutz test: Impact of collimator angle, phantom position, and field size

**DOI:** 10.1002/acm2.70000

**Published:** 2025-02-04

**Authors:** Weiliang Du

**Affiliations:** ^1^ Department of Radiation Physics The University of Texas MD Anderson Cancer Center Houston Texas USA

**Keywords:** quality assurance, radiation isocenter, Winston‐Lutz test

## Abstract

**Purpose:**

This study aimed to evaluate the impact of collimator angle, ball bearing (BB) phantom position, and field size on the accuracy of Winston‐Lutz (WL) test–derived radiation isocenters.

**Methods:**

WL tests were performed on four TrueBeam linear accelerators. Fifty‐six images (eight gantry angles multiplied by seven collimator angles) were acquired for each WL test. Images with different sets of collimator angles were used to compute the radiation isocenters. The resulting radiation isocenters were correlated with the collimator angles. Then, the BB position and radiation field size were varied for the subsequent WL tests. The calculated BB shifts were compared with the known shifts, and the radiation isocenters were compared between different field sizes.

**Results:**

The use of a single collimator angle led to errors of as much as 0.4 mm in the calculated radiation isocenters. Systematic differences were observed between the radiation isocenters derived with collimator angle 0° and those derived with 90° and/or 270°. A commonly used opposing collimator angle pair, 90° and 270°, resulted in a vertical 0.1 mm offset of the radiation isocenters toward the ceiling. Oblique opposite or mixed collimator angles yielded radiation isocenter errors less than 0.1 mm. The BB shifts derived from WL tests were less than 0.1 mm from the known shifts. The radiation isocenters varied by less than 0.1 mm between field sizes ranging from 2 × 2 cm^2^ to 20 × 20 cm^2^.

**Conclusions:**

Oblique opposing collimator angle pairs should be considered to minimize errors in localizing radiation isocenters. Uncertainty in BB positioning could be eliminated if the BB is used as a static reference point in space. The field size had no significant effect on the radiation isocenters. With careful design of WL test parameters and image processing, it is possible to achieve a precision of 0.1 mm in localizing radiation isocenters using WL tests.

## 1 INTRODUCTION

Localization of the isocenter of a C‐arm linear accelerator (linac) has been an important part of quality assurance (QA) programs for external beam radiotherapy. The isocenter is generally defined as the point around which the gantry, collimator, and the treatment couch rotate.^1^ Depending on whether mechanical devices or radiation fields are used to track these rotational axes, the resulting intersection of these axes is called the mechanical isocenter or the radiation isocenter. Historically, front pointers are used to localize the mechanical isocenter; however, this method is manual, time‐consuming, and not feasible for routine use.^2^ Numerous other devices have been developed to help identify the linac isocenter, including light field crosshairs, laser systems, surface tracking systems, on‐board imagers, ceiling mounted x‐ray imagers, etc. These devices can provide convenient surrogates for the isocenter, but they do not provide the isocenter by themselves. In fact, the coincidence of these devices with the isocenter must be checked periodically to ensure spatial accuracy.^3^ The importance of localization of the linac isocenter, especially the radiation isocenter, has been emphasized in numerous practice guidelines for medical physicists.^1^, ^3^, ^4^

Two methods are commonly used to localize the radiation isocenter: star (or spoke) shots and the Winston‐Lutz (WL) test.^5^, ^6^ The star‐shot technique is used to track the radiation field motion by exposing a film at different angles. This technique is two‐dimensional (2D) in nature; that is, for each film, only one rotation (gantry, collimator, or couch) is studied. In contrast, the WL test can track the radiation fields in 3D space. A ball‐bearing (BB) phantom is exposed at different gantry, collimator, and couch angles in a single WL test. By analysis of the positions of the BB center and radiation field centers, the rotational axes and subsequently the radiation isocenter are derived relative to the BB position.^7^, ^8^ For this reason, the WL test method is preferred over the star‐shot technique in evaluating the radiation isocentricity of C‐arm linacs, as described in the American Association of Physicists in Medicine (AAPM) Medical Physics Practice Guideline (MPPG) 8b.^1^

Although the concept of the WL test is simple, that is, measuring the concentricity of radiation fields and a BB phantom, the implementation method varies in practice. First, “BB position” may have different meanings. In the early practice of cranial stereotactic radiosurgery, the BB simulated the radiosurgery targets by being set to their stereotactic ordinates.^6^ When WL tests are used for linac isocenter QA nowadays, the BB can be moved so that it is centered inside all radiation fields to be imaged.^9^, ^10^ A compromise is usually made to find the optimal BB position to account for the wobble of radiation fields during gantry and collimator rotations and mechanical imperfection during couch rotation. In this scenario, the optimized BB position is regarded as the radiation isocenter. Alternatively, the BB is positioned at the mechanical isocenter via in‐room lasers, light field crosshairs, etc., or at the imaging isocenter via imaging.^8^, ^11^, ^12^ Then, the BB offset from the radiation isocenter is interpreted as the misalignment between the corresponding point (mechanical or imaging isocenter) and the radiation isocenter. Another method is to use the BB as a reference point in the 3D space.^7^, ^13^, ^14^, ^15^ In this method, the BB is positioned at an arbitrary stationary point as long as it is close to the linac isocenter. This method has gained popularity because of its rapid setup time and the potential to eliminate BB positioning errors. The drawback is that the calculated BB offset from the radiation isocenter is no longer interpreted as the mechanical (or imaging) versus radiation isocenter coincidence.

Second, different combinations of gantry, collimator, and couch angles have been used in WL tests. For gantry angles, the four cardinal angles are often employed. For collimator angles, the most common choices are a pair of opposing collimator angles^10^, ^13^, ^16^, ^17^, ^18^, ^19^, ^20^ or a single collimator angle of 0°.^21^, ^22^, ^23^, ^24^, ^25^, ^26^ The use of opposite collimator angles enables the cancellation of collimator misalignment; however, residual systematic errors may exist due to gravitational effects on the collimator.^27^ For couch angles, 90° or 45° intervals are generally used. In some studies, the couch rotation was not considered; that is, couch rotation was excluded in the localization of radiation isocenters.^12^, ^14^, ^28^ Third, the radiation field sizes in WL tests can vary significantly, from 1 × 1 cm^2^ to 24 × 24 cm^2^.^12^, ^26^, ^28^, ^29^, ^30^ The small fields are convenient for a visual check of the concentricity of BB and radiation fields, whereas the large fields allow more accurate localization of radiation field edges. Moreover, the radiation field edges are affected by the tongue‐and‐groove design of the multi‐leaf collimator (MLC). Thus, the choice of the field size may impact the radiation isocenter location determined with WL tests.

In this study, the author assessed the accuracy of using WL tests to localize radiation isocenters. Specifically, the effects of WL test parameters, namely the BB position, collimator angle, and radiation field size, on the WL test‐derived radiation isocenters were investigated. The BB position was used merely as a stationary reference point in 3D space and did not need to be placed at the mechanical or imaging isocenter.^13^, ^15^ The goal of this study was not to determine the coincidence between the radiation isocenter and mechanical or imaging isocenter. Rather, the WL tests were used for high‐precision localization of the radiation isocenters. For collimator angles, the author investigated the impact of the commonly used single angle 0° and paired opposite angles, quantifying systematic errors from using these angles with the aim of finding the optimal set of collimator angles. To study the field sizes, the author used the MLC to form radiation fields of various sizes and performed WL tests using each of the field sizes. Knowledge of the radiation isocenter dependence on the WL test parameters should help advance the precision limit of radiation isocenter localization.

## 2 METHODS

### 2.1 Phantom and linacs

**FIGURE 1:**
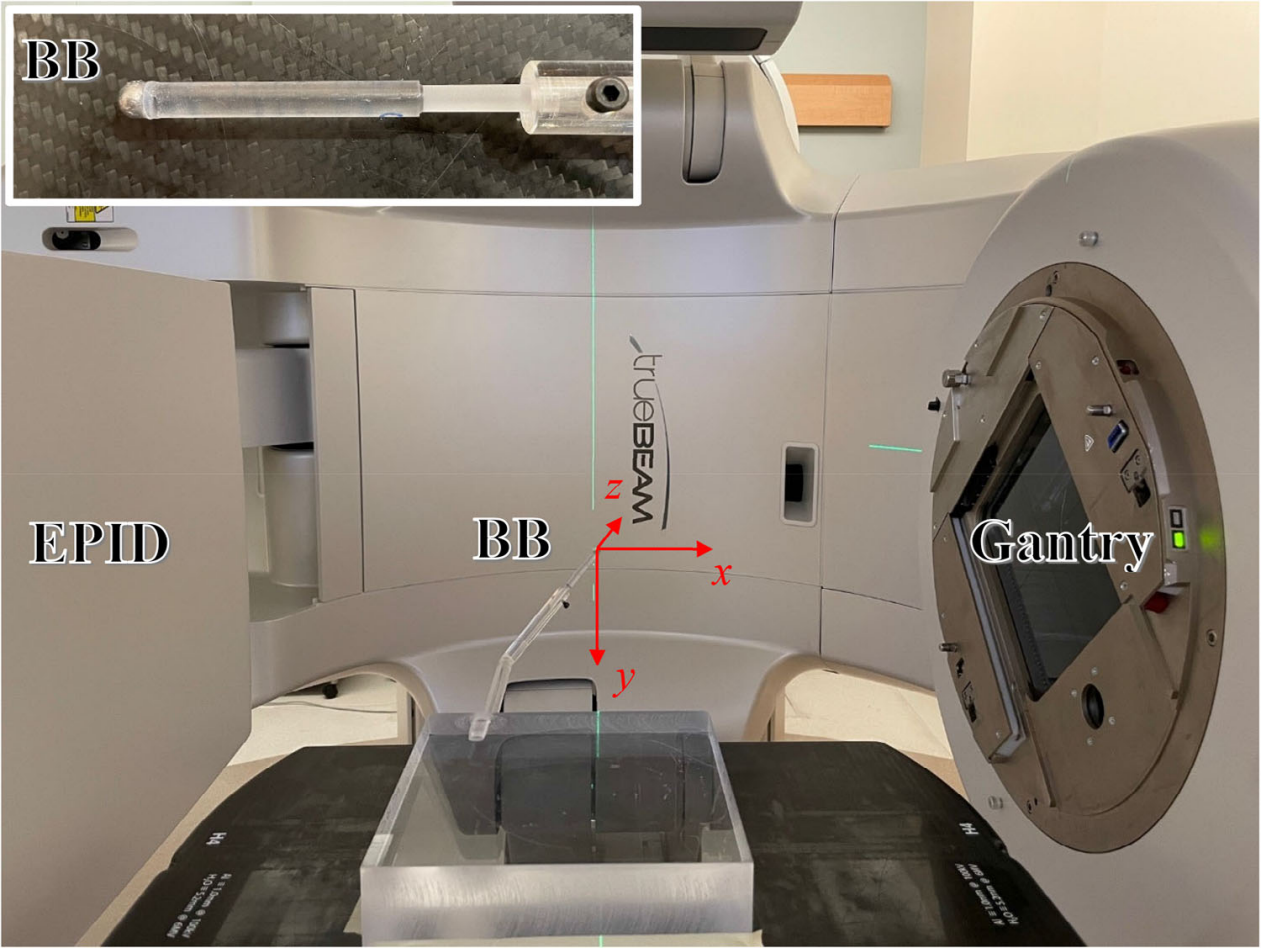
The ball bearing (BB) phantom for Winston‐Lutz (WL) tests and the x‐y‐z coordinate system. The red arrows indicate the positive directions. The inset shows the tungsten BB glued on the tip of an acrylic rod. The gantry was set at 90° in this picture.

The phantom was simply a tungsten BB 6.5 mm in diameter glued on an acrylic rod, which was screwed into an acrylic base block (Figure 1). The BB was positioned near the linac isocenter (within ± 2 mm) using the room lasers. The BB served as a static reference point in the space. The BB offset from the radiation isocenter was determined at the end of the WL analysis, as described in Section 2.3 below. The base block was taped onto the couch and kept stationary during the entire image acquisition, except for the measurements specified in Section 2.4 below.

Measurements were done on four TrueBeam linacs (Varian Medical Systems, Palo Alto, CA). The Varian IEC 601‐2‐1 scale was used for the definition of gantry angle and collimator angle. The x‐, y‐, and z‐axes were defined by the static in‐room coordinate system with the origin set at the BB center. The positive directions pointed to the right side (+x) when facing the gantry, to the floor (+y), and to the gantry (+z). The TrueBeam linacs in this study were equipped with a Millennium 120‐leaf MLC and an aS1200 EPID. The EPID physical resolution was 0.336 × 0.336 mm^2^ at the imager plane. With a source‐to‐imager distance of 150 cm, the resolution was scaled to 0.224 × 0.224 mm^2^ at the linac isocenter.

### 2.2 Image acquisition

Megavolt images of the BB phantom were acquired in TrueBeam service mode. A 10 × 10 cm^2^ MLC field was loaded for all image acquisitions except for those described below in Section 2.5. The secondary jaws of the TrueBeam were set at 15 × 15 cm^2^.

Two types of WL tests (long and short) were performed. The long WL test was used to study the collimator effect on the radiation isocenter localization, where a large number of gantry/collimator combinations were employed. Eight gantry angles (225°, 270°, 315°, 0°, 45°, 90°, 135°, and 180°) were used. At each gantry angle, seven collimator angles (225°, 270°, 315°, 0°, 45°, 90°, and 135°) were used. The couch angle was fixed at 0°. The couch rotation was not considered in this study. A total of 56 images were acquired in approximately 20 min for each long WL test.

The short WL test, which is more efficient, was used to study the effects of phantom position and field size. Four gantry angles (270°, 0°, 90°, and 180°) were employed. At each gantry angle, two opposite collimator angles (225° and 45°) were used. This gantry/collimator angle set was selected after review of the results in Section 3.1. A total of eight images were acquired for each short WL test in approximately 5 min.

### 2.3 Effect of collimator angles

To calculate the radiation isocenter, each WL image was analyzed to locate the BB center and the radiation field center. A beam's central ray (BCR) was constructed in the x‐y‐z space that was perpendicular to the image plane and passed through the radiation field center. Then, the intersection of all BCRs was determined to be the radiation isocenter. Mathematically, the radiation isocenter was computed as the point in 3D space that minimized the sum of squared distances from the point to each BCR.^27^

Because the exact radiation isocenter location is never known, it was hypothesized that the radiation isocenter can be approached by analyzing as many WL images or BCRs as possible. In other words, the systematic and random errors of the resulting radiation isocenter would gradually diminish as the number of BCRs increases. For each long WL test, consisting of 56 images, the BCR data were sampled into 31 different subsets, each representing a unique combination of gantry angles and collimator angles (Table 1). The number of gantry angle samples (N_gantry_) was either 4 or 8. The number of collimator angles (N_collimator_) varied from 1 to 7. The number of BCRs (N_BCR_) was the product of N_gantry_ and N_collimator_. The BCRs within each subset were used to calculate a unique radiation isocenter location. N_BCR_ ranged from 4 to 56 among different subsets.

**TABLE 1:** Winston‐Lutz (WL) data subsets used to localize the radiation isocenters. The original WL data consisted of 56 images acquired at eight gantry angles multiplied by seven collimator angles.

Subset	Gantry angles (°)	Collimator angles (°)	Collimator group	N_BCR_
1	[270,0,90,180]	0	C = 0	4
2	[270,0,90,180]	90	C = 90|270	4
3	[270,0,90,180]	270	C = 90|270	4
4	[225,315,45,135]	0	C = 0	4
5	[225,270,315,0,45,90,135,180]	0	C = 0	8
6	[225,270,315,0,45,90,135,180]	90	C = 90|270	8
7	[225,270,315,0,45,90,135,180]	270	C = 90|270	8
8	[270,0,90,180]	[270,90]	C = 90&270	8
9	[270,0,90,180]	[225,45]	C = Mixed	8
10	[270,0,90,180]	[315,135]	C = Mixed	8
11	[225,315,45,135]	[270,90]	C = 90&270	8
12	[225,315,45,135]	[225,45]	C = Mixed	8
13	[225,315,45,135]	[315,135]	C = Mixed	8
14	[270,0,90,180]	[270,0,90]	C = Mixed	12
15	[225,315,45,135]	[270,0,90]	C = Mixed	12
16	[225,270,315,0,45,90,135,180]	[270,90]	C = 90&270	16
17	[225,270,315,0,45,90,135,180]	[225,45]	C = Mixed	16
18	[225,270,315,0,45,90,135,180]	[315,135]	C = Mixed	16
19	[270,0,90,180]	[225,315,45,135]	C = Mixed	16
20	[225,315,45,135]	[225,315,45,135]	C = Mixed	16
21	[270,0,90,180]	[225,315,0,45,135]	C = Mixed	20
22	[225,315,45,135]	[225,315,0,45,135]	C = Mixed	20
23	[225,270,315,0,45,90,135,180]	[270,0,90]	C = Mixed	24
24	[270,0,90,180]	[225,270,315,45,90,135]	C = Mixed	24
25	[225,315,45,135]	[225,270,315,45,90,135]	C = Mixed	24
26	[270,0,90,180]	[225,270,315,0,45,90,135]	C = Mixed	28
27	[225,315,45,135]	[225,270,315,0,45,90,135]	C = Mixed	28
28	[225,270,315,0,45,90,135,180]	[225,315,45,135]	C = Mixed	32
29	[225,270,315,0,45,90,135,180]	[225,315,0,45,135]	C = Mixed	40
30	[225,270,315,0,45,90,135,180]	[225,270,315,45,90,135]	C = Mixed	48
31	[225,270,315,0,45,90,135,180]	[225,270,315,0,45,90,135]	C = Mixed	56

The 31 subsets were divided into four groups. Group 1 (C = 0) had a single collimator angle of 0°. Group 2 (C = 90|270) had a single collimator angle of either 90° or 270°. Group 3 (C = 90&270) used a pair of collimator angles, 90° and 270°. Group 4 (C = Mixed) had a mixture of collimator angles 0°, 90°, 270°, and/or oblique angles 45°, 135°, 225°, and 315°. To compare the 31 radiation isocenters, a reference radiation isocenter was calculated by averaging all radiation isocenters in Group 4. This averaged radiation isocenter was not regarded as the “true” radiation isocenter, but instead used as a reference for estimating the accuracy of the calculated radiation isocenters.

### 2.4 Effect of phantom position

To investigate the effect of BB position on radiation isocenter localization, the author moved the BB to different positions and ran a short WL test at each BB position. For each short WL test, the gantry angles were (270°, 0°, 90°, and 180°) and the collimator angles were (225° and 45°). The measurements were done in the following steps:

1. For the first WL test, the couch position was recorded as T_0_. The BB center position relative to the WL radiation isocenter was calculated as (BB–Iso)_0_ by analyzing the images of the first WL test.
2. Three random numbers were generated within the range of –2.5 to 2.5 mm. These random numbers were added to the x‐y‐z coordinates of T_0_ to yield T_1_. Then, T_1_ was used to move the couch to the next position. The couch movement was carried out in TrueBeam service mode.
3. Another WL test was performed and analyzed. The new BB center relative to the radiation isocenter was then calculated as (BB–Iso)_1_.
4. Steps 2 and 3 were repeated 10 more times, each time with new random numbers generated, that is, moving the BB to a new position.
5. The couch was moved back to the initial position T_0_, and the final WL test was performed.

At the end of measurements, the couch position T_i_ and the BB position relative to radiation isocenter (BB–Iso)_i_ were obtained, where index i ranged from 0 to 12. The changes of T_i_ and (BB–Iso)_i_ from their initial values T_0_ and (BB–Iso)_0_ were calculated as ΔT_i_ and Δ(BB–Iso)_i_, respectively. In essence, ΔT_i_ were the known BB shifts from its initial position, and Δ(BB–Iso)_i_ were the BB shifts determined with the WL tests. Ideally, there was an exact correspondence between ΔT_i_ and Δ(BB–Iso)_i_ if the following conditions were all met: (1) the radiation isocenter remained at the same position in 3D space for all WL tests, (2) the BB was precisely localized to the radiation isocenter in each WL test, and (3) mechanical movement of the couch (along with the BB) was precise. In practice, any differences between ΔT_i_ and Δ(BB–Iso)_i_ would indicate the imperfections in satisfying these three conditions. The paired data were fit with a linear model, Δ(BB–Iso)_i_ = a + k·ΔT_i_, where a and k were the intercept and slope of the fit, respectively.

### 2.5 Effect of field size

The effect of field size was studied on Linac 1. The side length of the MLC square field was varied to 2, 3, 5, 6, 10, 15, and 20 cm, respectively. The secondary jaws were set to 25 × 25 cm^2^, that is, outside the MLC fields. For each field size, the short WL tests were repeated five times. The radiation isocenters were computed from these WL tests and compared to the averaged positions.

One‐way analysis of variance (in MATLAB, MathWorks, Natick, MA) was run to detect if the field size was a factor causing a significant difference in the resulting radiation isocenters. A *p*‐value of 0.05 or less indicated statistical significance.

### 2.6 Comparison with a commercial software

To validate the algorithms for radiation isocenter localization, five WL tests were performed and the images were analyzed with both the in‐house software and the SunCHECK Winston‐Lutz Isocenter QA software (Sun Nuclear, Melbourne, FL). For each WL test, the gantry angles were (270°, 0°, 90°, and 180°). The other parameters are listed below.

1. WL‐1: MLC field size = 2 × 2 cm^2^, energy = 6 MV, collimator = 0°.
2. WL‐2: MLC field size = 3 × 3 cm^2^, energy = 6 MV, collimator = 0°.
3. WL‐3: MLC field size = 3 × 3 cm^2^, energy = 15 MV, collimator = 0°.
4. WL‐4: MLC field size = 3 × 3 cm^2^, energy = 6 MV, collimator = 45°.
5. WL‐5: MLC field size = 10 × 10 cm^2^, energy = 6 MV, collimator = 0°.

For each WL test, four images were processed independently by the in‐house software and by SunCHECK software. The 2D offsets between the BB and BCR, or (BB‒BCR), were calculated for each image. The 3D offsets between the BB and radiation isocenter, or (BB–Iso), were calculated for each WL test. The differences between the in‐house results and those calculated by SunCHECK were quantified.

## 3 RESULTS

### 3.1 Effect of collimator angle

**FIGURE 2:**
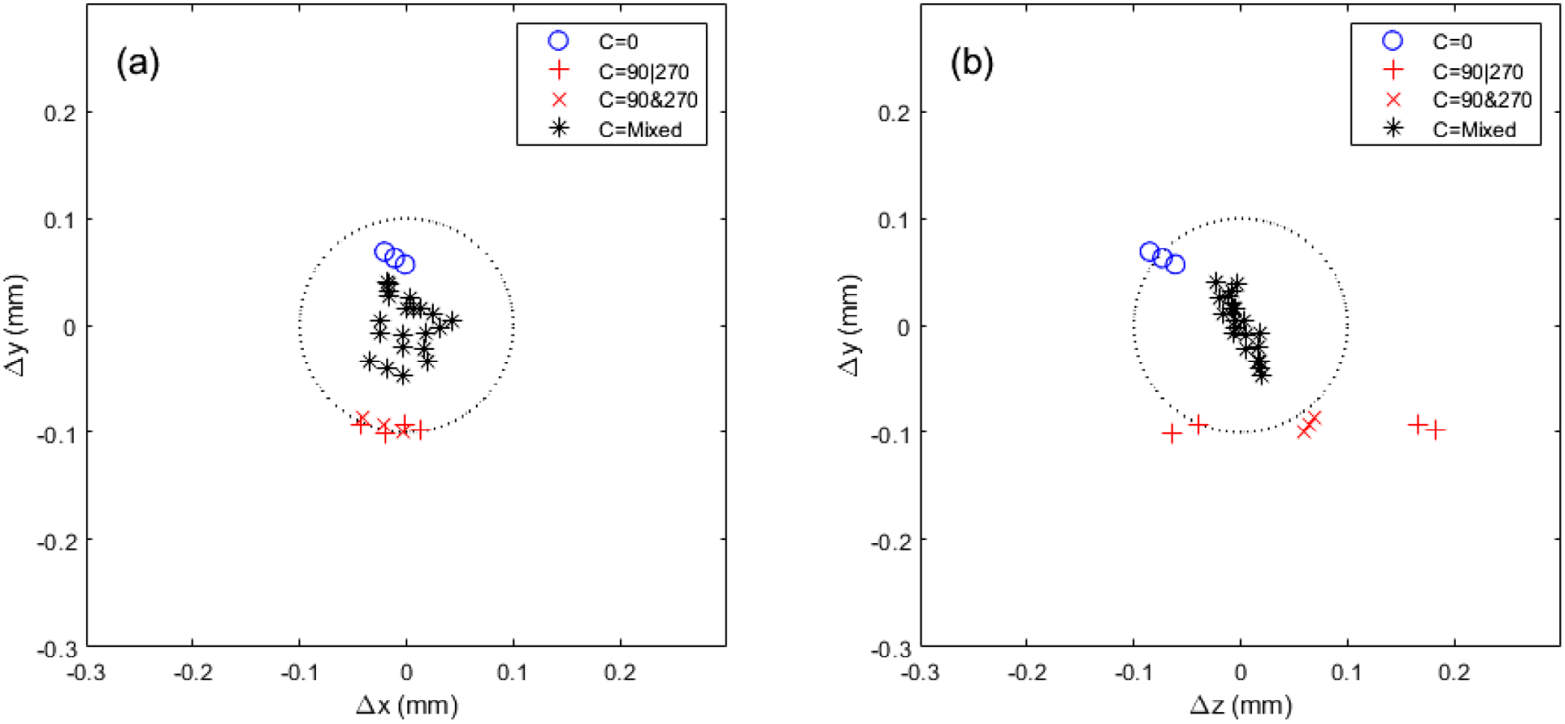
Radiation isocenters derived from different sampling schemes in Table 1. Data were obtained from one Winston‐Lutz (WL) test on Linac 1. The center of the plot was the reference radiation isocenter (i.e., an average of C = Mixed group). (a) Radiation isocenters in x‐y plane. (b) Radiation isocenters in z‐y plane. The dotted circle was centered at (0, 0) and had a radius of 0.1 mm.

Figure 2 shows the radiation isocenters calculated from one WL test on Linac 1. The majority of these radiation isocenters belonged to the C = Mixed group (Table 1) and were clustered within a circle of 0.1 mm radius. The average position of the C = Mixed radiation isocenters served as the reference radiation isocenter, to which all calculated radiation isocenters were compared. The radiation isocenters in the C = 0 group were 0.16 mm larger in y than the radiation isocenters in both the C = 90|270 and C = 90&270 groups. The z coordinates varied by 0.23 mm within the C = 90|270 group (Figure 2b). When the opposite pair C = 90&270 was used, the resulting radiation isocenter z coordinates were <0.1 mm from the reference radiation isocenter.

**FIGURE 3:**
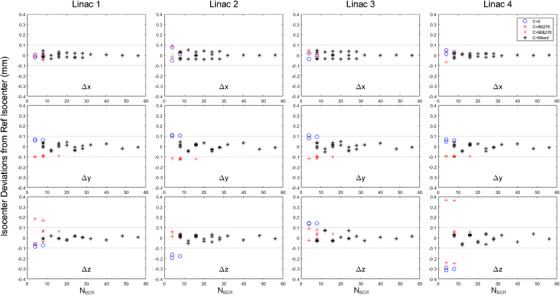
Radiation isocenter deviations from the reference radiation isocenter on four TrueBeam linacs. For each linac, 31 radiation isocenters were calculated from the sampling schemes in Table 1. The reference radiation isocenter was the average of radiation isocenters in the C = Mixed group. The dotted lines are ± 0.1 mm from the reference radiation isocenter.

The radiation isocenter data from the four linacs are shown in Figure 3. The x coordinates of all radiation isocenters were within 0.1 mm from the reference radiation isocenters (i.e., an average of C = Mixed). The y and z coordinates of the radiation isocenters started with > 0.1 mm deviations at the lowest N_BCR_ values and then converged to the reference radiation isocenters as N_BCR_ increased. Indeed, the radiation isocenter derived from 56 BCRs was nearly identical to the reference radiation isocenter. In the y‐direction, there was a clear difference between the C = 0 group and the C = 90|270 and C = 90&270 groups. The difference was approximately 0.2 mm on all four linacs, and the C = 0 radiation isocenters had a larger y (i.e., towards the floor) than those with C = 90|270 and C = 90&270. This consistent difference was much smaller but still existent within the C = Mixed group. For example, the N_BCR_ = 40 radiation isocenters contained the collimator angle 0° but not 90° or 270°. Thus, the N_BCR_ = 40 radiation isocenters had slightly larger y than the reference radiation isocenters. Similarly, the N_BCR_ = 48 radiation isocenters had slightly smaller y due to the inclusion of C = 90&270 but not C = 0 data.

The z coordinates showed inconsistent differences between C = 0 and C = 90|270 groups. With a single collimator angle, the radiation isocenters could be as much as 0.3–0.4 mm from the reference radiation isocenter (see Linac 4). The C = 0 radiation isocenters had positive z coordinates for Linac 3, and negative z coordinates for the other three linacs, indicating that the z offsets of the radiation isocenters were machine‐specific and collimator angle specific. For group C = 90&270, where opposite collimator angles were used, all radiation isocenters were <0.1 mm from the reference radiation isocenter in the z‐direction; the same did not hold for the y‐direction. The y coordinates of C = 90&270 radiation isocenters were consistently at approximately ‒0.1 mm from the reference radiation isocenter, even with N_BCR_ = 16 (i.e., with eight gantry angles).

The C = Mixed group had 21 different combinations of gantry and collimator angles, with N_BCR_ ranging from 8 to 56. Figures 2 and 3 show that all radiation isocenters in the C = Mixed group were within 0.1 mm from the reference radiation isocenter in all three dimensions. Table 2 shows the radiation isocenter deviations from the reference radiation isocenters for the long (N_BCR_ = 56, index 31 in Table 1) WL test versus the short (N_BCR_ = 8, index 9 in Table 1) WL test. The 3D absolute deviations, averaged across the four linacs, were 0.014 and 0.045 mm for the long and short WL tests, respectively. The image acquisition time was 20 min for the long WL test and 5 min for the short WL test. Given the small (<0.1 mm) radiation isocenter deviations, the author chose to use the short WL test for further repeated measurements in studying the effects of phantom position and field size.

**TABLE 2:** Radiation isocenter deviations from the reference radiation isocenter. Long WL tests included all 56 images in radiation isocenter calculation. Short WL tests used only eight images.

		Δ_x_ (mm)	Δ_y_ (mm)	Δ_z_ (mm)	Δ (mm)
Long WL Test	Linac 1	−0.004	−0.009	0.004	0.010
	Linac 2	0.001	−0.010	−0.008	0.013
	Linac 3	0.000	−0.008	0.014	0.017
	Linac 4	0.002	−0.010	−0.012	0.016
Short WL Test	Linac 1	0.044	0.004	−0.005	0.044
	Linac 2	0.034	0.006	0.025	0.043
	Linac 3	0.046	0.003	−0.031	0.055
	Linac 4	−0.019	0.011	0.030	0.037

Abbreviation: WL, Winston‐Lutz.

### 3.2 Effect of phantom position

**FIGURE 4:**
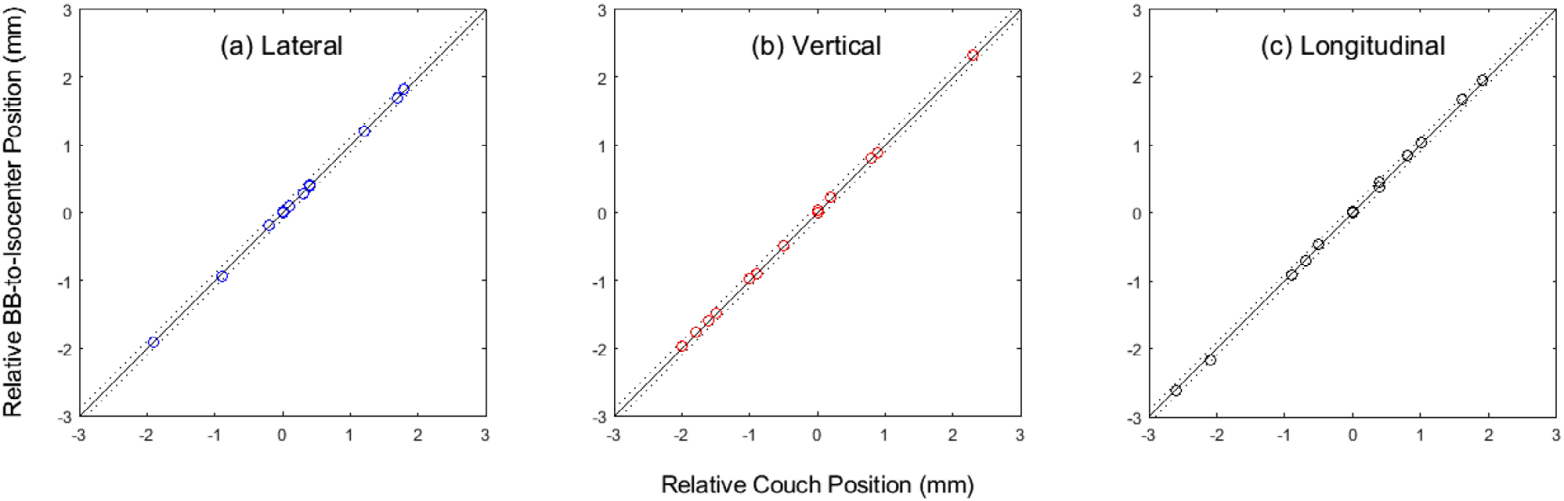
The ball bearing (BB)‐to‐isocenter shifts, or Δ(BB–Iso), derived from Winston‐Lutz (WL) tests versus the known couch shifts, ΔT. The solid diagonal lines are Δ(BB–Iso) = ΔT. The dotted lines are 0.1 mm above or below the solid lines.

Figure 4 shows the effect of shifting the BB phantom in radiation isocenter localization. The horizontal axis was couch shifts, or ΔT, and the vertical axis was WL test‒determined BB shifts, or Δ(BB–Iso). There was an excellent agreement between Δ(BB–Iso) and ΔT, in the range of ‒2.5 to 2.5 mm. All data points fell close to the diagonal line, Δ(BB–Iso) = ΔT, within ± 0.1 mm of uncertainty. The average absolute difference between Δ(BB–Iso) and ΔT was 0.043 mm, and the maximum absolute difference was 0.071 mm. The linear fit results of Δ(BB–Iso) versus ΔT are shown in Table 3. Again, the values of the slope and *R* ^2^ suggested a strong linear relationship between Δ(BB–Iso) and ΔT.

**TABLE 3:** Linear fit of manual couch shifts, or ΔT, and WL test‒calculated BB shifts, or Δ(BB–Iso).

	Slope (k)	Intercept (a)	*R* ^2^
Lateral (x)	1.0036	−0.005	0.9998
Vertical (y)	0.9981	0.015	0.9999
Longitudinal (z)	1.0202	0.012	0.9997

### 3.3 Effect of field size

**FIGURE 5:**
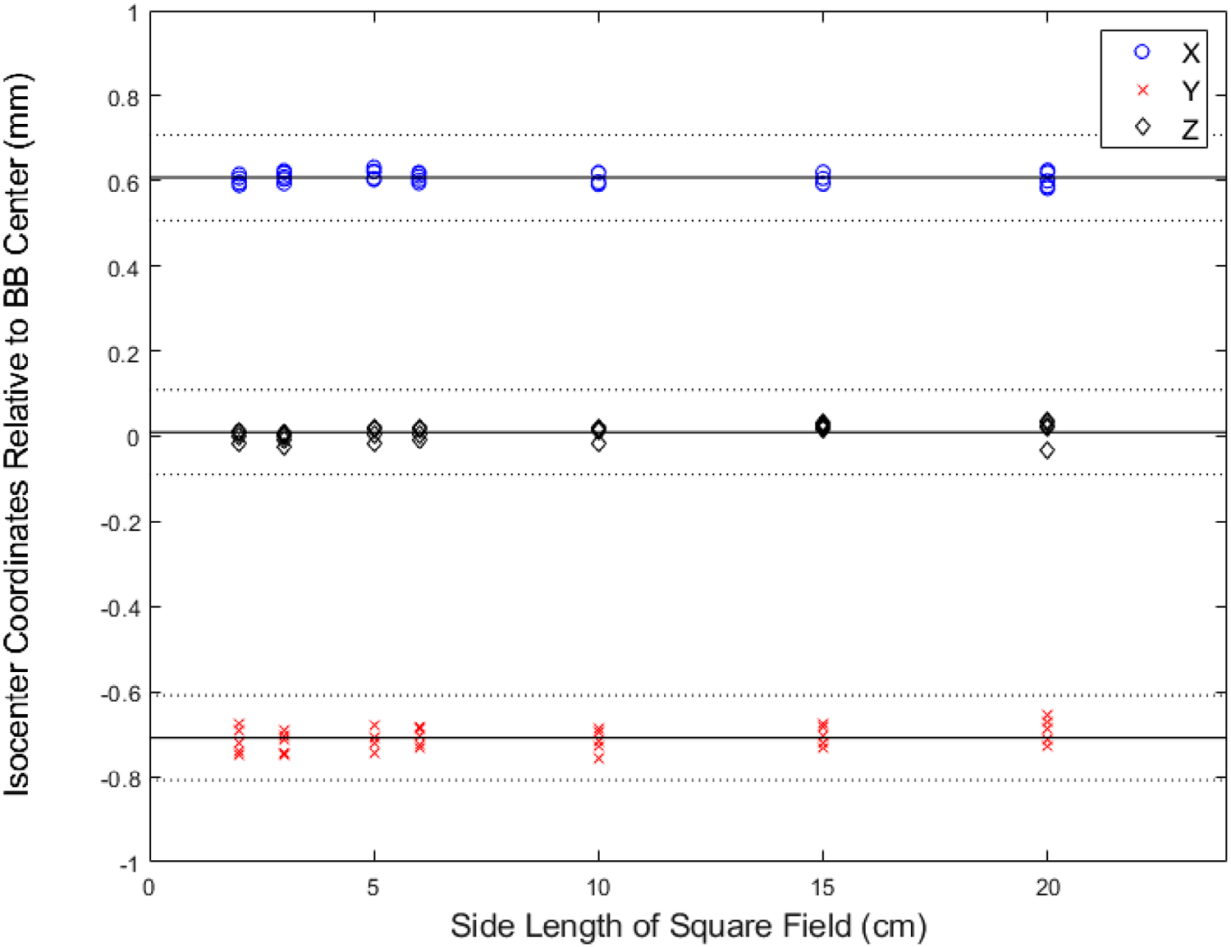
Radiation isocenter‐to‐ball bearing (BB) positions determined with the Winston‐Lutz (WL) tests using different MLC field sizes. The solid lines are the average radiation isocenter coordinates from all seven field sizes. The dotted lines are ± 0.1 mm from the solid lines.

The dependence of WL test radiation isocenters on the MLC field sizes is illustrated in Figure 5. In all three dimensions, the radiation isocenters at different field sizes were well within ± 0.1 mm from the averaged radiation isocenter location. The *p*‐values of the one‐way analysis of variance were 0.64, 0.67, and 0.20 for the x‐, y‐, and z‐directions, respectively. Thus, no significant differences were found in the WL test‐determined radiation isocenters with varying field sizes.

### 3.4 Comparison with SunCHECK software

Table 4 shows the differences in the BB positions relative to the BCRs or the radiation isocenter, calculated with the in‐house software versus SunCHECK software. The 2D (BB–BCR) offsets are presented only for the WL‐4 test as an example. (BB–BCR) varied as the gantry was rotated. The in‐house software and SunCHECK software computed (BB–BCR) with a difference within 0.2 mm. Among the 20 images for the five WL tests, the mean absolute difference in (BB–BCR) was 0.14 mm with a standard deviation of 0.06 mm. The 3D (BB–Iso) offsets are also displayed in Table 4. The mean absolute difference in (BB–Iso) was 0.10 mm with a standard deviation of 0.04 mm.

**TABLE 4:** Comparison of 2D offsets, or (BB–BCR), and 3D offsets, or (BB–Iso), computed with the in‐house software and the SunCHECK software.

		In‐house (mm)	SunCHECK (mm)	Δ (mm)	|Δ| (mm)
**(BB**–**BCR) for WL‐4**	Gantry = 270°	(−0.82, 0.65)	(−0.80, 0.50)	(−0.02, 0.15)	0.15
	Gantry = 0°	(−0.62, 0.24)	(−0.59, 0.13)	(−0.03, 0.11)	0.11
	Gantry = 90°	(0.56, 0.34)	(0.71, 0.26)	(−0.15, 0.08)	0.17
	Gantry = 180°	(0.37, 0.70)	(0.45, 0.54)	(−0.08, 0.16)	0.18
**(BB**–**Iso)**	WL‐1	(−0.59, 0.29, 0.89)	(−0.66, 0.34, 0.81)	(0.07, −0.05, 0.08)	0.12
	WL‐2	(−0.15, −0.10, −0.18)	(−0.14, −0.11, −0.23)	(−0.01, 0.01, 0.05)	0.05
	WL‐3	(−0.16, −0.12, −0.31)	(−0.16, −0.12, −0.37)	(0.00, 0.00, 0.06)	0.06
	WL‐4	(−0.49, 0.69, 0.48)	(−0.52, 0.75, 0.36)	(0.03, −0.06, 0.12)	0.14
	WL‐5	(−0.46, 0.27, 0.89)	(−0.57, 0.31, 0.86)	(0.11, −0.04, 0.03)	0.12

Abbreviations: BB, ball bearing; BCR, beam's central ray; WL, Winston‐Lutz.

## 4 DISCUSSION

In this study, the author first evaluated the effect of collimator angle on WL test–based radiation isocenter localization. A widely used opposing pair, 90° and 270°, yielded a vertically higher radiation isocenter (i.e., towards the ceiling) than that of the collimator angle 0°. The vertical difference of ∼0.2 mm was small, yet consistent between the four TrueBeam linacs studied. A similar difference was reported earlier on Varian Clinac machines.^27^ A possible cause is that the gravitational pull‐on MLC leaves at gantry angles 90° or 270° is the largest with a collimator angle 0° and the smallest with a collimator angle 90° or 270°. When intermediate collimator angles were used, the resulting radiation isocenters were in between the radiation isocenters derived from C = 0 and C = 90&270 (Figure 3). This finding indicates that the commonly used C = 90&270 collimator angles may not be optimal in terms of localizing the “average” radiation isocenter for all collimator angles. Other opposite pairs, such as C = 45&225 and C = 135&315, should be considered as potentially better alternatives.

Collimator misalignment is another major source of errors in radiation isocenter localization. While the radiation isocenter errors in the x‐ and y‐directions are alleviated with the use of opposing gantry angles, the collimator misalignment in the z‐direction does not cancel out with the gantry rotation. Thus, opposing collimator angles are still necessary to minimize the radiation isocenter errors in the z‐direction. In this study, when a single collimator was used, the error in z could be as much as 0.38 mm (Figure 3, Linac 4). The errors in z were quickly reduced to within 0.1 mm by simply using the opposite collimator angles.

In a previous study done on Varian Clinac machines, the radiation isocenters were localized with an accuracy of 0.2 mm by using opposite gantry and collimator angles.^27^ The current study confirmed the systematic vertical offsets of the radiation isocenters derived with C = 0 versus C = 90&270 on TrueBeam linacs. In addition, this study found that the localization accuracy of radiation isocenters can be further improved to the 0.1 mm level by using oblique opposite collimator angles or mixed collimator angles to minimize systematic errors.

The agreement of calculated and measured BB shifts in Figure 4 was remarkable. This kind of agreement required high precision (<0.1 mm uncertainty) of linac mechanical movements (couch translation, gantry rotation, collimator rotation, MLC leaf movement) and subpixel precision of computerized image analysis to calculate the radiation isocenters. Figure 4 suggested that the radiation isocenters remained at the same point (<0.1 mm uncertainty) in the 3D space despite the varied positions of the BB phantom. When Capaldi et al.^31^ tested their BB localization algorithm by intentionally moving the BB by known amounts, the linear fit of calculated BB offsets versus physical offsets yielded a slope of 1.14. Douglass and Keal^32^ compared the calculated BB shifts using deep learning WL analysis with the manual BB shifts. The slope was 1.046 and the absolute differences were on the order of a pixel size (∼0.25 mm). In contrast, the slopes of linearity in this work ranged from 0.998 to 1.020 and the maximal difference between calculated and manual BB shifts was 0.071 mm. Thus, the results in this work showed that BB and radiation isocenter positions were computed with a <0.1 mm uncertainty. In other words, the phantom setup errors had an effect of <0.1 mm on the accuracy of WL radiation isocenters in this study.

The field size was not found to affect the radiation isocenter location. The radiation isocenters generated with different field sizes were within 0.1 mm from the average location. The independence of radiation isocenter localization from field sizes is not surprising. Like collimator misalignment, any systematic shifts of the field centers when the field sizes are varied would cancel out with opposite collimator angles. Historically, small field sizes such as 2 × 2 cm^2^ were often used in film‐based WL tests for an easy visual check of the concentricity of radiation fields and the BB phantom. In the era of digital WL tests, small field sizes may hinder the accurate localization of BB and radiation field edges, especially when the BB is offset and close to the field edges. Computer algorithms such as thresholding and Canny edge detection may result in significant errors when the BB is close to the field edge.^30^, ^32^

In the current study, the author aimed at localizing the radiation isocenter with <0.1 mm of precision. Of note, the individual BCRs, from which the radiation isocenter was synthesized, had deviations from the radiation isocenter much larger than 0.1 mm. The wobble of the BCRs around the radiation isocenter, also referred to in the literature as the isocenter size, is one of the most important parameters in characterizing linac performance. Quantification of the isocenter size relies critically on the accurate localization of the radiation isocenter itself. Errors in radiation isocenter localization would directly propagate into the quantification of the isocenter size. Given the tight isocenter size on modern linacs, often on the order of 1 mm or less, there is a strong need for <0.1 mm of precision in radiation isocenter localization.

It was interesting to compare the present method with the independent, commercially available SunCHECK WL test method. Although the two methods employed very different algorithms to localize radiation isocenters for the same set of input images, the numerical results were comparable, as detailed in Table 4. In addition, when the SunCHECK‐calculated (BB–BCR) data were fed to the in‐house software, the (BB–Iso) results became identical (difference less than 0.01 mm) between the two methods. This indicates that the main difference between the two methods arises from the localization of BB and radiation fields in 2D images. The in‐house software used the Hough transform to localize the BB circle and the radiation field edges, while the SunCHECK software used a thresholding technique to segment the BB and the radiation fields. The thresholding technique may be sensitive to the choices of thresholds, the signals from the stem that support the BB, and non‐uniformity in image intensities. Nonetheless, the difference in the resulting radiation isocenters between the present method and the SunCHECK method was on the order of 0.1 mm.

One limitation of this work is the small sample size (4 linacs) and the evaluation of only one type of linac. The methods in this work can be used to evaluate other linacs; however, certain details may vary. For example, the 0.2 mm vertical difference between collimator angles C = 0 and C = 90&270 may be different on other linacs.

Another limitation is that this work dealt with the linac radiation isocenter, not an off‐axis point or multiple points, which have been the subject of recently emerged off‐axis WL tests. The role of the BB can be very different between at‐isocenter WL tests and off‐axis WL tests. In the former, the BB can be the surrogate of a stereotactic radiosurgery target, or it can be a static reference point in 3D space. When used as a static reference point, the BB position has no impact on the radiation isocenter localization (Figure 4). In off‐axis WL tests, however, the BB represents a stereotactic target at a certain distance from the isocenter. If the BB appears off from the radiation field centers, the offsets may indicate imperfect linac mechanical movements (of the gantry, collimator, or MLC), inaccurate stereotactic positioning of the BB, or both. Thus, it is conceptually difficult to use the BB as an arbitrary reference point in the off‐axis WL tests. The idea of using the BB as a reference point may not work in off‐axis WL tests.

## 5 CONCLUSIONS

The use of opposing collimator angles was critical to minimize the effect of collimator misalignment in radiation isocenter localization. Oblique opposite or mixed collimator angles should be considered to further minimize the systematic errors due to gravitational effects on the MLC leaves. The BB positions were accurately accounted for in the WL radiation isocenter analysis, as the radiation isocenter was localized with <0.1 mm of uncertainty and was independent of the BB position. Finally, the different MLC field sizes caused little change (less than 0.1 mm) in the resulting radiation isocenters. With careful selection of WL test parameters and suitable computer algorithms, a precision of 0.1 mm in localizing the linac radiation isocenter is attainable.

## AUTHOR CONTRIBUTIONS

**Weiliang Du**: Conceptualization; methodology; data collection; data analysis; investigation; writing and revising the manuscript.

## CONFLICT OF INTEREST STATEMENT

The author declares no conflicts of interest.
